# Effects of Dietary Kelp Enzymatic Hydrolysate Supplementation on Performance, Antioxidant Capacity, Immune Function, and Intestinal Health of Sows and Their Piglets

**DOI:** 10.3390/md24100354

**Published:** 2026-10-09

**Authors:** Can Zhang, Yuankai Qu, Lei Xue, Bo Cheng, Jianjun Zang

**Affiliations:** 1 National Feed Engineering Technology Research Center, College of Animal Science and Technology, China Agricultural University, No. 2 Yuanmingyuan West Road, Beijing 100193, China; b20233040380@cau.edu.cn (C.Z.);; 2 Weihai Shidai Marine Biotechnology Co., Ltd., Weihai 264319, China

**Keywords:** kelp enzymatic hydrolysate, reproductive performance, immunoglobulin, short chain fatty acid, sows, piglets

## Abstract

This study is the first to investigate the effects of dietary supplementation with kelp enzymatic hydrolysate (KEH) from day 85 of gestation to day 21 of lactation in sows. Twenty-four Landrace × Yorkshire sows were randomly assigned to four treatments: a control diet (0% KEH) and three experimental diets supplemented with 0.75%, 1.50%, or 3% KEH. Backfat thickness at 24 h postpartum increased linearly with KEH supplementation (*p* = 0.02), while the number of piglets born alive showed a quadratic response (*p* = 0.03). KEH linearly increased glutathione peroxidase activity in umbilical cord blood (*p* = 0.02) and quadratically affected catalase activity in 21-day-old piglets (*p* = 0.02), which peaked at 1.50% KEH. Concentrations of immunoglobulin A (IgA; *p* = 0.01) and M (IgM; *p* = 0.02) in umbilical cord blood were quadratically enhanced by KEH, peaking at 1.50% and at 0.75% KEH, respectively. Serum IgG in 7-day-old piglets and IgM and IgG in 21-day-old piglets were linearly and quadratically influenced by KEH (*p* < 0.05). Quadratic responses were observed in serum triiodothyronine (*p* = 0.03) and interleukin-1β (*p* < 0.01) in 7-day-old piglets, as well as in serum interleukin-6 in 21-day-old piglets (*p* < 0.01). KEH had linear and quadratic effects in interleukin-1β in 14- and 21-day-old piglets (*p* < 0.05). Quadratic tendencies were detected in fecal acetic acid concentration in sows (*p* = 0.09) and in fecal isobutyric acid (*p* = 0.06) and isovaleric acid (*p* = 0.07) content in piglets, whereas the fecal valeric acid concentration in piglets was quadratically affected (*p* = 0.02) by KEH. Intestinal *Firmicutes* and *Bacteroidota* were the dominant phyla, with *Lactobacillus* being the most dominant genus in both sows and piglets. In piglets, *Holdemanella* was positively correlated with several short-chain fatty acids (SCFAs). Overall, dietary 0.75–1.50% KEH during late gestation and lactation improved reproductive performance, immune and antioxidant capacity, inflammatory status, and intestinal SCFAs profiles in both sows and their offspring.

## 1. Introduction

Kelp, a large brown alga, is rich in polysaccharides, amino acids, minerals, vitamins and polyphenols. Enzymatic hydrolysis converts kelp into kelp enzymatic hydrolysate (KEH), which breaks down insoluble fibers and macromolecules into small molecules and improves the bioavailability of its core active component, fucoidan [1,2,3]. As a sulfated polysaccharide, fucoidan modulates intestinal microbiota by stimulating the proliferation of beneficial bacteria and inhibiting pathogens. Its microbial fermentation produces short-chain fatty acids (SCFAs), which improve intestinal barrier function, maintain immune homeostasis, regulate metabolism and relieve inflammation [4,5]. Laminarin, another major algal polysaccharide, is indigestible in the livestock small intestine, but it has been shown to enhance growth performance, antioxidant capacity, immunity and intestinal microecology in animals [6,7]. Nevertheless, the impacts of KEH on sow reproduction and progeny health remain largely unclear.

Late gestation is a nutritionally critical stage that determines maternal physiological state, metabolic efficiency and fetal development [8]. Rapid fetal growth triggers substantial physiological, metabolic and immunological changes in sows [9]. Suppressing inflammation and maintaining physiological homeostasis are critical for improving reproductive traits and gut microbial balance [10]. Maternal intestinal microbiota can be vertically transmitted to suckling piglets via the birth canal, colostrum and postnatal environment, shaping the initial gut colonization of newborns. Consequently, maternal gut dysbiosis impairs sows’ metabolism and immunity, and further hinders the growth and disease resistance of their offspring [11].

Oxidative stress is another key stressor for sows during late gestation [12]. Increased metabolic activity and fetal oxygen demand lead to excessive accumulation of reactive oxygen species (ROS). Excess ROS induce lipid peroxidation, which damages sow reproductive performance and reduces antioxidant enzyme activities in both sows and piglets [13,14]. Furthermore, sustained oxidative stress aggravates inflammatory responses, disturbs maternal metabolism and increases the risk of intestinal disorders in suckling piglets [15].

Nutritional regulation during gestation and lactation exerts profound effects on the health and productivity of sows and piglets. Marine-derived prebiotics and antioxidants serve as a safe, cost-effective and sustainable nutritional strategy. Featuring unique bioactivities, KEH fits well with the concept of sustainable feed development. Accordingly, this study was carried out to explore the effects of four graded KEH levels (0, 0.75%, 1.50% and 3%) supplemented in sow diets from day 85 of gestation to day 21 of lactation on reproductive performance, antioxidant status, immune function and intestinal health, and to determine its optimal dosage.

## 2. Results

### 2.1. Reproduction and Growth Performance of Sows and Their Piglets

In Table 1, dietary KEH supplementation influenced sow backfat thickness at 24 h postpartum (linear, *p* = 0.02; Figure S1; quadratic, *p* = 0.07). KEH also quadratically affected the number of piglets born alive per litter (*p* = 0.03, Figure S2) and tended to affect the average birth weight of piglets (quadratic, *p* = 0.09). However, no differences were observed in sow body weight, average daily feed intake, or total milk yield (*p* > 0.05).

### 2.2. Antioxidant Capacity in Serum of Sows and Piglets

As shown in Table 2, dietary KEH supplementation linearly increased glutathione peroxidase (GSH-Px) activity in umbilical cord blood (*p* = 0.02, Figure S3a) and tended to quadratically affect it (*p* = 0.08), but did not affect the activities of total superoxide dismutase (T-SOD), malondialdehyde (MDA), or catalase (CAT) (*p* > 0.05). In 21-day-old piglets, CAT activity showed a significant quadratic response to KEH supplementation (*p* = 0.02, Figure S3b), peaking at the 0.75% and 1.50% KEH levels and declining thereafter. No significant differences were observed in serum antioxidant indices on days 7 or 14 (*p* > 0.05).

### 2.3. Immunoglobulin Concentrations in Serum of Sows and Piglets

In Table 3, umbilical cord blood IgA (*p* = 0.01, Figure S4a) and IgM (*p* = 0.02, Figure S4b) concentrations showed quadratic response to increasing KEH levels, with the highest IgA and IgM levels observed at 1.50% and 0.75% KEH, respectively. In piglets, serum IgG at day 7 was affected by both linear (*p* = 0.02) and quadratic (*p* < 0.01) effects of KEH supplementation (Figure S4c). At day 14, KEH tended to linearly increase serum IgA (*p* = 0.07). At day 21, had serum IgM and IgG concentrations were affected by both linear and quadratic effects of KEH (*p* < 0.05, Figure S4d,e).

### 2.4. Thyroid Hormones and Inflammatory Cytokine Levels in Serum of Piglets

As shown in Table 4, there was quadratic response in serum triiodothyronine (T3) concentration (*p* = 0.03, Figure S5a) and interleukin-1β (IL-1β) level (*p* < 0.01, Figure S5b) at day 7. At days 14 and 21, IL-1β levels were affected by both linear and quadratic effects of KEH (*p* < 0.05, Figure S5c,d). Furthermore, at day 21, serum interleukin-6 (IL-6) concentration was quadratically affected by KEH supplementation (*p* < 0.01, Figure S5e).

### 2.5. Short-Chain Fatty Acids Contents in Feces of Sows and Piglets

In sow, fecal acetic acid (C2) concentration showed a quadratic tendency (*p* = 0.09), peaking at the 1.50% KEH level. However, dietary KEH supplementation did not affect the fecal concentrations of propionic acid, isobutyric acid, butyric acid, isovaleric acid, valeric acid, or total short chain fatty acids (TSCFAs) (*p* > 0.05). In piglets, KEH supplementation tended to affect fecal isobutyric acid (i-C4) (*p* = 0.06) and isovaleric acid (i-C5) (*p* = 0.07), and significantly influenced valeric acid concentration (C5) (*p* = 0.02, Figure S6) in quadratic response (Table 5).

### 2.6. Fecal Microbiota of Sows and Piglets

**Figure 1:**
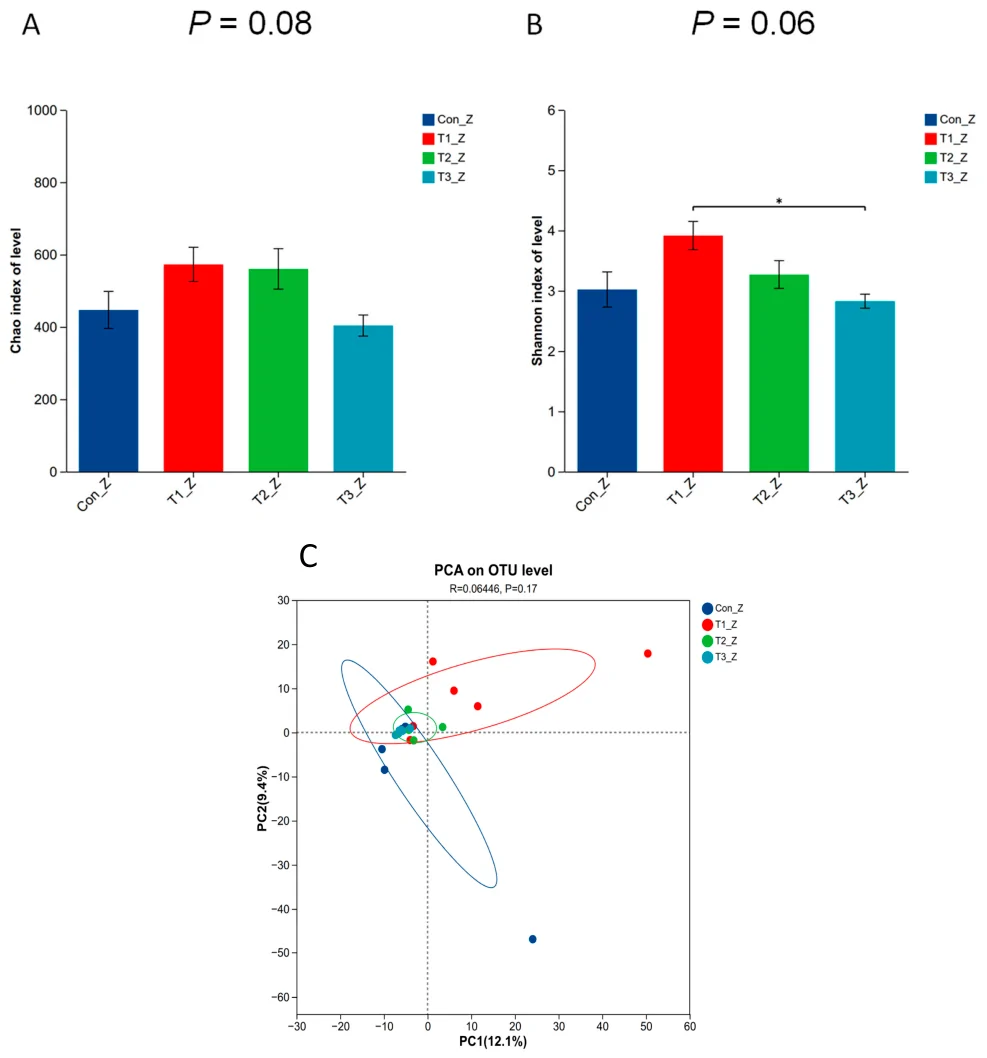
Diversity and richness of fecal bacterial communities in piglets. (**A**,**B**) Alpha diversity metrics. Chao (**A**) and Shannon (**B**) indexes for fecal microbiota of piglets. (**C**) Plot of principal component analysis (PCA). A shorter distance between the sample points denotes greater similarity of the bacteria (*n* = 6 for each group). * Indicates a tendency (*p* < 0.10). Abbreviations: Con_Z, T1_Z, T2_Z, and T3_Z represent the addition of 0%, 0.75%, 1.50%, and 3% KEH in the diet for sows, respectively.

**Figure 2:**
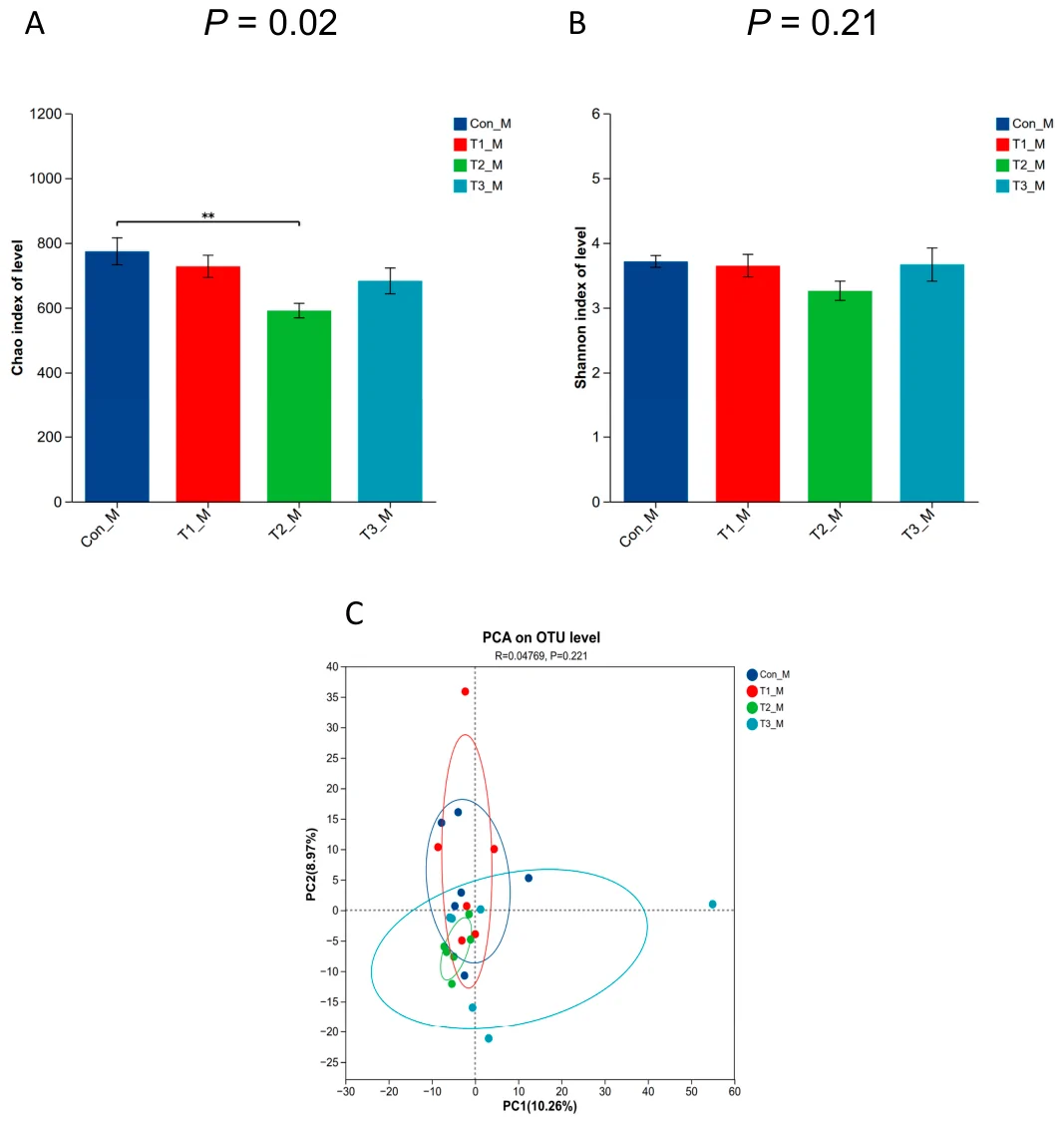
Diversity and richness of fecal bacterial communities in sows. (**A**,**B**) Alpha diversity metrics. Chao (**A**) and Shannon (**B**) indexes for fecal microbiota of sows. (**C**) Plot of principal component analysis (PCA). A shorter distance between the sample points denotes greater similarity of the bacteria (*n* = 6 for each group). ** indicates a significant difference (*p* < 0.05). Abbreviations: Con_M, T1_M, T2_M, and T3_M represent the addition of 0%, 0.75%, 1.50%, and 3% KEH in the diet for sows, respectively.

**Figure 3:**
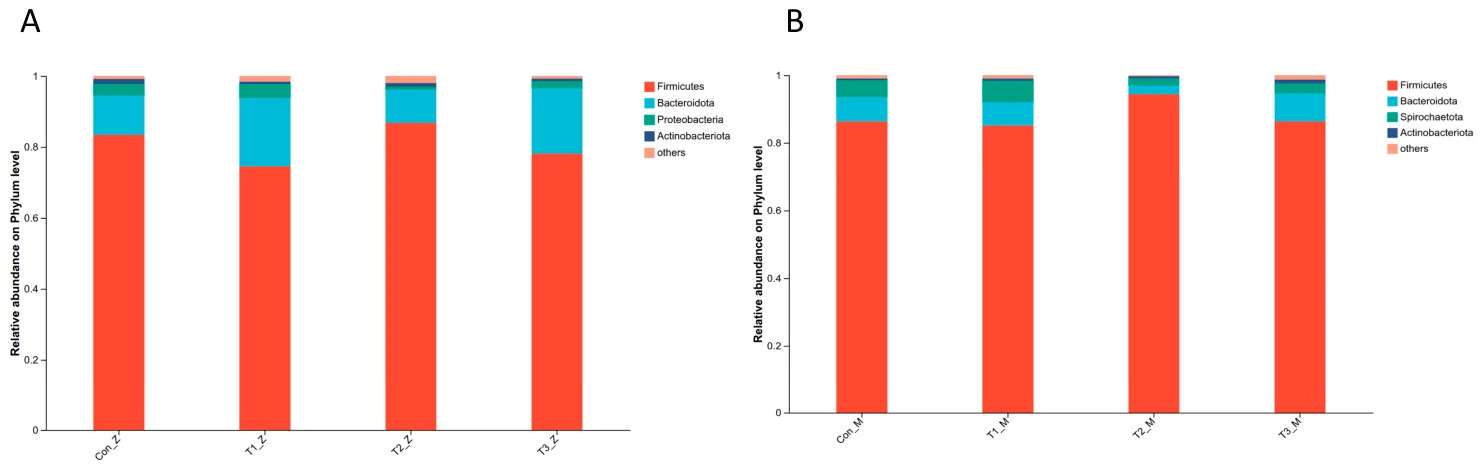
Relative abundance of fecal microbiota at the phylum level in piglets (**A**) and sows (**B**) (n = 6 per group). Abbreviations: Con_Z, T1_Z, T2_Z, and T3_Z represent the addition of 0%, 0.75%, 1.50%, and 3% KEH in the diet for sows, respectively. Con_M, T1_M, T2_M, and T3_M represent the addition of 0%, 0.75%, 1.50%, and 3% KEH in the diet for sows, respectively.

**Figure 4:**
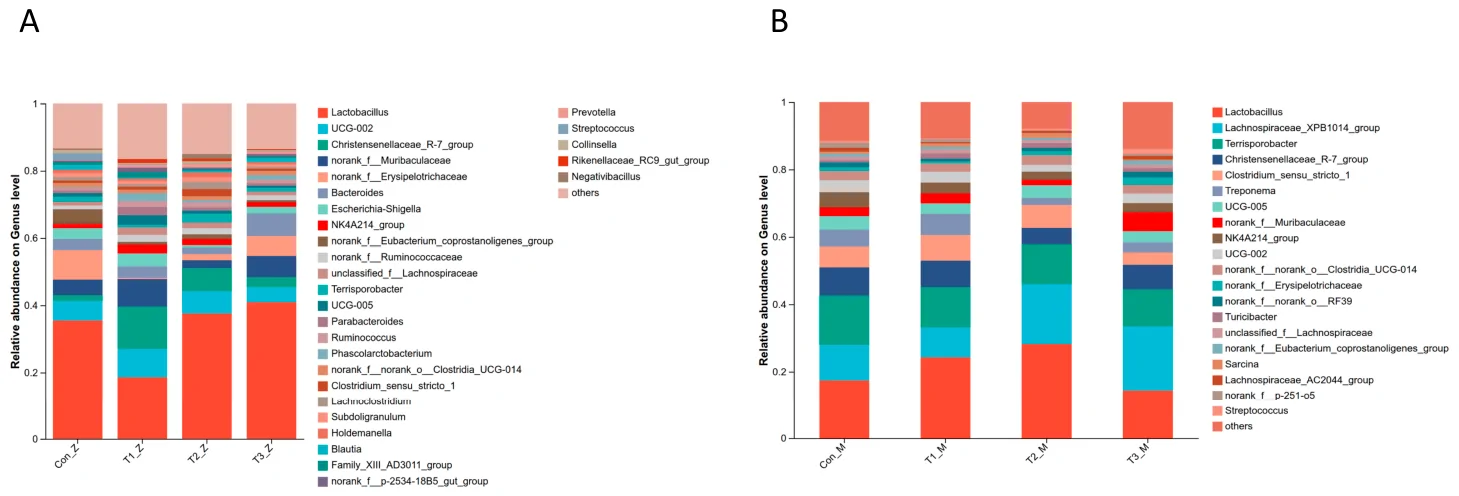
Relative abundance of fecal microbiota at the genus level in piglets (**A**) and sows (**B**) (n = 6 per group). Abbreviations: Con_Z, T1_Z, T2_Z, and T3_Z represent the addition of 0%, 0.75%, 1.50%, and 3% KEH in the diet for sows, respectively. Con_M, T1_M, T2_M, and T3_M represent the addition of 0%, 0.75%, 1.50%, and 3% KEH in the diet for sows, respectively.

**Figure 5:**
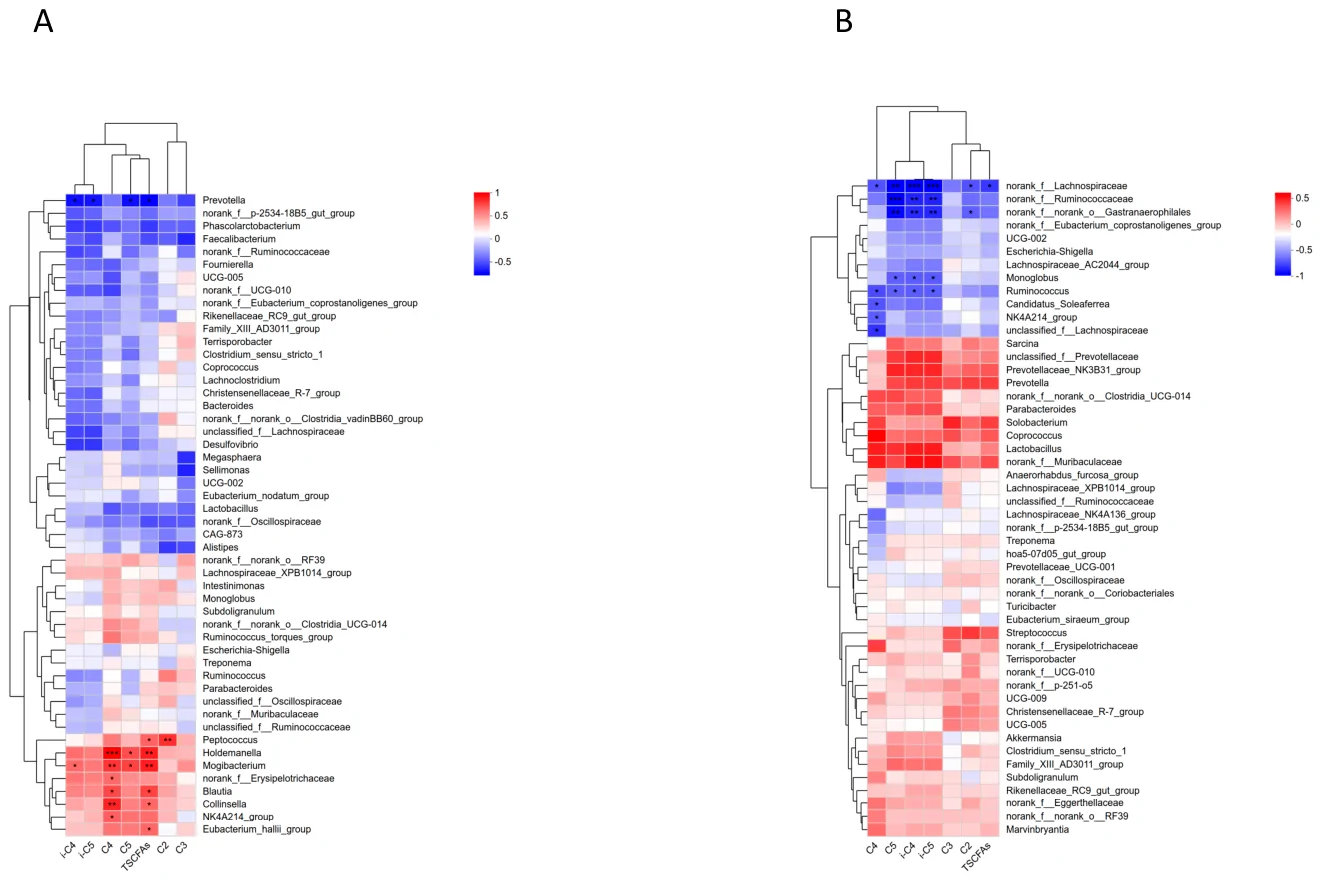
Correlation of fecal microbiota with SCFAs in piglet (**A**) and sow (**B**) feces (n = 6 for each group). * *p* ≤ 0.05, ** *p* < 0.01, *** *p* < 0.001. Abbreviations: C2, acetic acid; C3, propionic acid; C4, butyric acid; C5, valeric acid; i-C4, isobutyric acid; i-C5, isovaleric acid; TSCFAs, total short-chain fatty acids.

The effects of dietary KEH supplementation on the alpha diversity of fecal microbiota are shown in Figure 1 (piglets) and Figure 2 (sows). In piglets, EH supplementation tended to affect the Chao index (*p* = 0.08) and the Shannon index (*p* = 0.06), both of which initially increased and then decreased as the KEH level increased. Specifically, the Shannon index tended to be higher in the 0.75% KEH group than in the 3% KEH group (*p* < 0.10). In sows, KEH supplementation significantly affected the Chao index (*p* = 0.02), with the control group showing a significantly higher Chao index than that in 1.50% KEH group (*p* < 0.05). However, no significant differences were observed in the Shannon index among the sow groups (*p* = 0.21). Principal component analysis (PCA) of fecal microbiota at the OTU level showed substantial overlaps among the groups (Figure 1C and Figure 2C), and no significant differences in the overall microbial community structure were detected in either piglets (R = 0.084, *p* = 0.17) or sows (R = 0.047, *p* = 0.22). At the phylum level, the dominant bacterial phyla in piglet feces (Figure 3A) were *Firmicutes*, *Bacteroidota*, *Proteobacteria*, and *Actinobacteriota*, whereas those in sow feces (Figure 3B) were *Firmicutes*, *Bacteroidota*, *Spirochaetota*, and *Actinobacteriota*. At the genus level, *Lactobacillus* was the predominant genus in both piglet (Figure 4A) and sow feces (Figure 4B), although its relative abundance numerically differed among groups. Spearman correlation analysis at the genus level showed that C4 (*p* < 0.001), C5 (*p* < 0.05) and TSCFAs (*p* < 0.01) were positively correlated with *Holdemanella*; however, i-C5, i-C4, C5 and TSCFAs were negatively correlated (*p* < 0.05) with *Prevotella* in piglet feces (Figure 5A). In sow feces (Figure 5B), i-C5, i-C4 and C5 were negatively correlated with norank_f_Lachnospiraceae, norank_f_Ruminococcaceae, and norank_f_norank_o_Gastranaerophilales(*p* < 0.01).

## 3. Discussion

Dietary KEH supplementation increased backfat thickness at 24 h postpartum, which may be attributed to improved nutrient utilization. Research by Rattigan et al. [16] demonstrated that laminarin supplementation upregulates the expression of glucose transporters and the long-chain fatty acid transporter FABP2 in the jejunum., and enhanced expression of transporters for sugars, fatty acids, peptides, and amino acids is known to improve energy and protein utilization in pigs [16]. Therefore, it is possible that this mechanism underlies the greater backfat thickness observed in KEH-supplemented sows at 24 h postpartum. However, backfat thickness should not be excessive, as backfat thickness ≥23 mm has been reported to promote excessive placental lipid deposition and exacerbate placenta oxidative stress [17], and embryonic oxidative stress is a major cause of developmental arrest [18,19]. The increased number of piglets born alive observed with KEH supplementation in this study is consistent with a previous report that dietary laminarin, a component of KEH, improves sow reproductive performance [20]. Laminarin, a β(1 → 3)-glucan with β(1 → 6)-branches, is a carbohydrate reserve in brown algae [19] that has been shown to enhance the developmental competence of porcine embryos by preventing oxidative stress-induced injury, preserving mitochondrial function, and upregulating pluripotency- and differentiation-related genes [21]. Total litter size is primarily determined by fertilization rate and early embryonic survival [22]; accordingly, dietary intervention during late gestation, as in the present study, did not influence this parameter.

In addition to exerting adverse effects on early embryos, excessive accumulation of reactive oxygen species (ROS) induces systemic oxidative stress and chronic inflammation in pregnant sows, which can potentially compromise fetal redox homeostasis [23]. In this study, GSH-Px activity in umbilical cord blood increased linearly with dietary KEH supplementation, which could help reduce the levels of toxic ROS [24]. Dietary seaweed polysaccharide supplementation has been reported to enhance antioxidant enzyme activity and free radical scavenging capacity [25]. Fucoidan, another component of KEH, can effectively scavenge ROS, suppress lipid peroxidation, and inhibit oxidative stress-induced cell death, thereby mitigating oxidative damage in cells and tissues [26]. Selenium is also a major component in KEH: it is incorporated into polypeptide chains aselenocysteine, and proteins containing selenocysteine are classified as selenoproteins [27,28]. GSH-Px is the main selenoprotein, and previous studies have consistently shown that maternal dietary organic Se supplementation during late or entire gestation could both enhance the antioxidant capacities of sows, which is transfer this to their offspring [29,30]. Given that the placenta and umbilical cord mediate the transfer of nutrients, gases, and metabolites between the mother and the developing fetus [31,32], the increased GSH-Px activity in the umbilical cord blood associated with KEH supplementation may partly account for the increased number of born alive per litter. In 21-day-old piglets, CATactivity peaked in the 0.75% and 1.5% KEH groups. Although this elevated CAT activity suggests that maternal KEH supplementation may bolster the antioxidant system of offspring, it is noteworthy that higher doses of fucoidan (2000 mg/kg) may trigger inflammation and metabolic disorders [33]. Thus, while fucoidan holds potential as a novel antioxidant, careful attention must be paid to the dosage used.

IgA serves as the first line of immune defense on mucosal surfaces and in bodily fluids [34]. In mammals, IgM predominantly exists as pentamers with low affinity but high avidity, providing non-specific binding that constitutes an innate first line of defense against invading pathogens [35]. IgG, the dominant antibody in the secondary immune response, mainly exists as a monomer and serves as a biomarker of systemic immune activation of a [36,37]. In the present study, umbilical cord blood IgA and IgM concentrations peaked at 1.50% KEH and 0.75% KEH, respectively, which may be attributed to the anti-inflammatory and immunomodulatory properties of algal compounds in KEH. Specifically, algal polysaccharides can bind to bacterial cell wall components, increasing membrane permeability and inhibiting pathogens [38,39]. Moreover, laminarin modulates cytokine production via the MAPK and JAK/STAT pathways, thereby activating immune responses while mitigating chronic inflammation [40]. During pregnancy, maternal IgG is transferred to the fetus via the placenta and umbilical cord [41], and protects piglets against environmental infections before weaning [42]. In this study, KEH supplementation regulated serum IgG concentration in piglets, with both linear and quadratic effects detected at 7 days and 21 days of age.

T4 and T3 regulate a suite of critical physiological processes throughout embryogenesis and larval development [43], including metabolic rate, thermogenesis, cardiac function, stress response, and development [44]. In this study, dietary KEH supplementation was associated with a quadratic response in serum T3 concentration in 7-day-old piglets. Iodine in KEH is essential for thyroid function and serves as a key substrate for thyroid hormone biosynthesis, representing a potential mechanism underlying the elevated serum T3 concentrations [45]. IL-6 is a key biomarker of the systemic inflammatory response syndrome [46], whereas IL-10 is an immunosuppressive cytokine that induces immune tolerance and plays a role in preventing premature birth [47]. TNF-α is a potent pro-inflammatory cytokine that negatively impacts embryonic development [48] and excessive production of TNF-α, IL-6 and IL-1β can lead to severe inflammation and damage to healthy cells, contributing to autoimmune disorders [49]. For neonatal piglets, whose immune systems are immature, the balance between pro- and anti-inflammatory cytokines is closely linked to their resistance to pathogens and growth performance.

Compared with the control group, dietary KEH supplementation decreased serum IL-1β concentrations in piglets at 7, 14, and 21 days of age, with the lower levels at 7 days observed in the 0.75% and 1.50% KEH groups. Dai et al. (2021) [50] found that pigments in brown algae inhibit the production of IL-1β and IL-6 in HaCaT keratinocytes. Additionally, fucoidan fermentation products inhibit pro-inflammatory cytokines, including IL-6, IL-1β, and TNF-α, in lipopolysaccharide (LPS)-stimulated macrophages [51]. Fucoidan exerts a core anti-inflammatory effect by downregulating these key pro-inflammatory cytokines and inhibiting the NF-κB and MAPK signaling pathways in multiple organ systems [52]. Taken together, these findings further confirm the anti-inflammatory potential of brown algae-derived components in neonatal piglets and extend the understanding of their immunomodulatory effects in young livestock. The sustained reduction of IL-1β by KEH supplementation throughout critical neonatal period is particularly important for piglets with underdeveloped immune barriers.

The hindgut microbiome, dominated by bacteria but also comprising bacteria, yeasts, fungi, and protozoa, plays a central role in degrading cellulose, hemicellulose, and pectin into SCFAs [53,54], principally acetic acid, propionic acid, butyric acid, isobutyric acid, valeric acid, and isovaleric acid. Beyond serving as energy substrates, SCFAs interact with immune cells and modulate tissue metabolism [55], signal partly through G-protein-coupled receptor [56], promote eosinophil apoptosis [57], and suppress downstream IL-5 and IL-13 release from eosinophils [58]. In present study, supplementation with 0.75% and 1.50% KEH increased acetic acid concentrations in sow feces and elevated isobutyric acid, isovaleric acid and valeric acid in piglet feces, while decreasing serum IL-1β piglets. Given that acetate modulates immune function and oxidative stress [59], these findings provides a plausible mechanistic link between KEH supplementation and the observed serum immune and antioxidant responses. Notably, inclusion of 1.50% KEH was associated with the highest serum IgG concentration in piglets on day 7, whereas peak concentrations of serum IgM and CAT activity were attained on day 21. Collectively, the KEH-induced increase in fecal acetate may be a key intermediary factor-enhancing humoral immunity and the attenuation of oxidative stress in piglets.

Significant differences were observed in bacterial diversity, but not the richness, in sow feces, whereas both richness and diversity tended to shift in piglet feces. This tendency may be attributed to fucoidan, the core bioactive component of KEH, which promotes the growth of beneficial bacteria, thereby increasing cecal SCFA concentrations [60] and regulating intestinal microbial homeostasis [61]. Such maternal dietary effects may be transmitted to offspring, potentially explaining the parallel patterns observed in sows and their piglets. Increasing evidence indicates that maternal diet during pregnancy can modify offspring microbiota composition and intestinal development over the long-term [62].

Many algae polysaccharides resist digestion by host enzymes and reach the colon intact, where they serve as prebiotic substrates that selectively enrich gut microbes and their activities [63]. Algae are therefore thought to act indirectly via the microbiota, with the postbiotic response reflected in increased production of SCFAs and other beneficial metabolites from microbial fermentation of algal prebiotics [64]. This mechanistic interpretation is strongly supported by the correlation analysis in the present study. At the genus level, fecal *Prevotella* in piglets was significantly negatively correlated with SCFAs, whereas *Holdemanella* showed a strong positive correlation with SCFAs. Succinate, one metabolite associated with *Prevotella* [65], has recently been identified as a dual-function metabolite in intestinal inflammation [66,67]. In addition, *Holdemanella* has been reported to be significantly positively correlated with volatile fatty acid metabolism in early weaned pigs [68], which is consistent with the present results. Thus, dietary supplementation with algal polysaccharides may selectively promote *Holdemanella* and thereby enhance SCFA production and intestinal health in piglets. In sows, i-C4, i-C5, and C5, which are typically derived from microbial fermentation of proteins/amino acids, were significantly negatively correlated with butyrate-producing bacteria (norank_f_Lachnospiraceae, norank_f_Ruminococcaceae, and norank_f_norank_o_Gastranaerophilales). These relationships suggest that KEH supplementation may reduce amino acid fermentation in the sows hindgut and indirectly shift microbial metabolism toward carbohydrate utilization by butyrate-producing bacteria, thereby lowering i-C4, i-C5, and C5 production.

Although several measured parameters reached statistical significance, the absolute magnitude of these changes was remained modest. These findings should therefore be regarded as preliminary evidence of a potential association rather than definitive proof of substantial production benefits. Further large-scale trials across parities and under varying environmental and management conditions are warranted to verify the practical implications and generalizability of these effects.

## 4. Materials and Methods

### 4.1. Animal Ethics

The experimental protocol was approved by the Institutional Animal Care and Use Committee of China Agricultural University (Beijing, China; approval No. AW70506202-1-07).

### 4.2. Animals, Diets and Experimental Design

A total of 24 Landrace × Yorkshire multiparous sows at day 85 of gestation with similar body weight (average 215.6 kg), parity (average 5.29) and backfat thickness (average 17.59 mm) were randomly assigned to four dietary treatments. Each treatment consisted of 6 replicated crates, each housing one sow. Sows had free access to water throughout the experiment. Pregnant or lactating sows in the control groups were fed the basal diet without KEH supplementation (0% KEH), whereas sows in the treatment groups were fed the corresponding basal diet supplemented with 0.75%, 1.50%, or 3% KEH (*w*/*w* relative to the basal diet). Within each feeding phase, the basal diet formula was identical among all treatments, and KEH was added on top of the basal diet without replacing any ingredient. KEH was prepared from fresh kelp through physical crushing, low-temperature enzymatic hydrolysis, separation, concentration, and drying. The enzymatic hydrolysis was performed using a composite enzyme preparation consisting of cellulase, pectinase, protease, and alginatease. The ratio of the enzyme complex to kelp (dry weight) was 1–3% (E/N), and the hydrolysis was conducted at 40–45 °C for 20–24 h. The resulting hydrolysate mainly contained seaweed oligosaccharides (≥80 g/kg), seaweed polysaccharides (≥40 g/kg), and betaine (≥800 mg/kg). Notably, the degree of polymerization (DP) of these seaweed oligosaccharides was mainly concentrated in the range of 2 to 6, which are known to exhibit relatively high biological activity. The high proportion of these low-DP oligosaccharides in the final product indicates that the polysaccharide components were substantially and deeply hydrolyzed. The chemical composition of KEH is shown in Table 6. The basal diets for sows in the late gestation and lactation stages were designed in line with the recommendations outlined in [69], and the ingredient lists as well as nutrient profiles of these basal diets are provided in Table 7. The experimental period lasted from day 85 of gestation to day 21 of lactation. Sows were individually housed in a temperature-controlled room (22–26 °C). From day 85 of gestation until parturition, all sows were fed 3.0 kg/d of diet, divided into three equal meals at 05:30, 10:00, and 15:00, in crates (2.1 m × 0.65 m). On day 110 of gestation, sows were moved to farrowing pens (2.1 m × 1.5 m). On the day of farrowing, each sow received 0.5 kg of the lactation diet; thereafter, the daily feed allowance was increased by 1.0 kg per day until ad libitum access was achieved. Suckling piglets had access to creep feed throughout the 21-day nursing period and were kept under optimal ambient temperature using infrared heat lamps. Routine management and immunization procedures followed the standard guidelines of the pig farm.

### 4.3. Sample Collection

On day 85 of gestation, backfat thickness (BF) was measured at the P1 position (6.5 cm from the last rib to the midline of the back) on the left side of each sow using a backfat meter (Mylab Touch Vet, Esaote S.p.A., Genoa, Italy). This measurement was used to assess body condition and ensure homogeneous group allocation. BF and body weight (BW) were recorded at 24 h postpartum and on the first day after weaning, and the changes in BF and BW during lactation were calculated.

Uneaten feed was weighed and removed every morning, and actual feed intake of each sow was calculated as the difference between feed offered and feed refused, from which average daily feed intake (ADFI) was determined. Total milk yield of individual sows on day 21 of lactation was estimated using the formula: total milk yield (kg) = piglet average daily gain (ADG) × litter size × lactation days × 4 [70].

Litter size at birth, number of piglets born alive per litter, and number of piglets with normal birth weight (>1.0 kg) were recorded. The BW of each piglet was measured at birth. Within 24 h after farrowing, piglets were cross fostered within each treatment group to standardize litter size to approximately 12 piglets per litter. Piglet BW was recorded after cross-fostering and on days 7, 14, and 21 of lactation, and ADG and weaning survival rate were subsequently calculated.

After expulsion of the placenta, approximately 10 mL of umbilical cord blood was collected from the blood-filled area of the umbilical cord. Blood samples were also collected from the inferior vena cava of piglets on days 7, 14, and 21 after birth. All blood samples were immediately centrifuged at 3000× *g* for 15 min at 4 °C to obtain serum. Fresh fecal samples from sows and piglets were collected on days 7, 14, and 21 postpartum. All samples were stored at −80 °C until analysis.

### 4.4. Sample Analysis

Serum levels of total superoxide dismutase (T-SOD), malondialdehyde (MDA), catalase (CAT), glutathione peroxidase (GSH-Px), immunoglobulin A (IgA), IgM, IgG, interleukin-1β (IL-1β), IL-6, IL-10, and tumor necrosis factor-α (TNF-α) were determined using commercial kits (Nanjing Jiancheng Bioengineering Institute, Nanjing, China) according to the manufacturer’s instructions. Serum concentrations of triiodothyronine (T3) and thyroxine (T4) were measured by radioimmunoassay.

For short-chain fatty acid (SCFA) analysis, approximately 1 g of fecal sample was placed into a 10 mL centrifuge tube, mixed with 8 mL of diluent and 200 μL of internal standard solution, and thoroughly vortexed. After ultrasonication for 30 min, the mixture was centrifuged at 3000 r/min for 10 min. The supernatant was filtered through a 0.22-μm filter membrane into a sample vial. SCFAs, including acetic, propionic, butyric, isobutyric, valeric, and isovaleric acids, were quantified using a gas chromatography system (Agilent Technologies, Santa Clara, CA, USA).

Total DNA was extracted from fecal samples using the E.Z.N.A.^®^ Stool DNA Kit (Omega Bio-Tek, Norcross, GA, USA) following the manufacturer’s protocol. DNA quality was assessed by 2% agarose gel electrophoresis. The V3–V4 hypervariable regions of the 16S rRNA gene were amplified by polymerase chain reaction (PCR) using genomic DNA from sow and piglet feces as templates. The primers used were 338F (5′-ACTCCTACGGGAGGCAGCAG-3′) and 806R (5′-GGACTACHVGGGTWTCTAAT-3′). The PCR program was as follows: initial denaturation at 95 °C for 3 min; 27 cycles of denaturation at 95 °C for 30 s, annealing at 55 °C for 30 s, and extension at 72 °C for 45 s; and a final extension at 72 °C for 10 min. After purification, PCR amplicons were pooled in equimolar concentrations and subjected to high-throughput sequencing on the Illumina MiSeq platform (Illumina, San Diego, CA, USA). Raw Illumina fastq files were quality-filtered using Fastp (version 0.19.6). Operational taxonomic units (OTUs) were clustered at 97% sequence similarity using UPARSE (version 11). Taxonomic annotation was performed against the Silva database using the RDP Classifier with a 70% confidence threshold.

### 4.5. Data Analysis

Data on sow reproductive performance and piglet growth performance were analyzed using one-way analysis of variance (ANOVA) with SPSS 27.0 (IBM Corp., Armonk, NY, USA). Before ANOVA, normality of residuals and homogeneity of variances were assessed using the Shapiro–Wilk and Levene’s tests, respectively. If the assumptions for ANOVA were violated, data were log- or square-root transformed prior to analysis, and the transformed data were subsequently subjected to ANOVA. Orthogonal polynomial contrasts were used to assess linear and quadratic effects of increasing KEH supplementation levels. All data are presented as mean and standard error of the mean (SEM). A *p*-value < 0.05 was considered statistically significant, and 0.05 ≤ *p* ≤ 0.10 was considered a tendency. For the 16S rRNA sequencing results of microorganisms, statistical analysis and graphing were performed using R 3.3.1. Alpha diversity was calculated with MOTHUR 1.30.2. Bacterial diversity was evaluated using the Shannon and Simpson indices, and bacterial richness was evaluated using the Chao1 and ACE indices. Inter-group differences in alpha diversity indices were tested using the *t*-test, with Welch’s t-test applied when variances were unequal. Welch’s t-test was also applied to compare species abundance between the two groups. High-dimensional biomarkers were identified using LEfSe. First, the Kruskal–Wallis H test was used to identify taxa with significant differences in abundance. Subsequently, Linear Discriminant Analysis (LDA) was employed to estimate the contribution of each taxon’s abundance to the differential effect.

## 5. Conclusions

This study indicates that dietary supplementation with 0.75–1.50% KEH during late gestation and lactation improves reproductive performance, antioxidant capacity, immune function, endocrine metabolism, and gut health in both sows and their offspring. The bioactive components of KEH appear to function as prebiotics, modulating hindgut microbiota by enriching beneficial bacteria and suppressing pathogenic bacteria, thereby enhancing SCFA production, which may subsequently promote immunoglobulin secretion and antioxidant enzyme activities. In addition, KEH may facilitate the maternal-to-offspring transfer of thyroid hormones while restraining excessive pro-inflammatory cytokine production. These findings support maternal KEH supplementation as a feasible nutritional strategy with potential health benefits for sows and their piglets.

## Figures and Tables

**Table 1:** Effects of the KEH supplementation on the performance of the sows and piglets.

Items	Levels				SEM	*p*-Value	
	0%	0.75%	1.50%	3%		Linear	Quadratic
Average of parity, *n*	5.17	5.33	5.33	5.33	0.52	0.32	0.62
Backfat thickness, mm						.	
Day 85 of gestation	17.50	17.67	17.50	17.67	0.82	0.96	1.00
Postpartum 24 h	13.60	18.50	17.25	18.17	0.64	0.02	0.07
After weaning	14.60	16.83	15.75	15.67	0.90	0.25	0.52
Backfat thickness change during lactation	1.00	−1.67	−1.50	−2.50	0.51	0.33	0.30
Body weight, kg							
Postpartum 24 h	254.3	241.3	240.4	244.0	4.81	0.51	0.68
Weaning	249.7	246.9	234.1	243.4	4.44	0.76	0.94
Body weight change during lactation	−4.57	5.57	−6.25	−0.63	2.02	0.54	0.69
Average daily feed intake (lactation), kg/d	5.29	5.52	6.49	5.80	0.17	0.35	0.29
Total milk yield, kg	221.3	325.2	257.0	265.1	14.01	0.62	0.18
Litter size at birth, n	12.17	16.00	13.75	13.83	0.74	0.57	0.42
Live born size per litter, n	10.17	15.33	13.50	12.83	0.59	0.32	0.03
Normal birth-weight piglets, n	9.33	12.17	11.75	11.33	0.58	0.29	0.20
Litter weight at birth, kg	15.48	20.22	16.96	17.00	0.86	0.85	0.33
Average birth weight, kg	1.55	1.33	1.26	1.48	0.05	0.61	0.09
Litter size after cross-fostering, n	11.67	11.33	12.25	12.00	0.28	0.46	0.75
Weaned piglets, n	10.33	11.00	11.50	11.00	0.26	0.29	0.32
Weaning survival rate, %	88.89	93.39	94.35	91.82	1.83	0.54	0.52
Average weight after cross-fostering, kg	1.52	1.35	1.31	1.43	0.06	0.57	0.35
Average weight on day 7, kg	2.41	2.29	2.47	2.56	0.09	0.40	0.57
Average weight on day 14, kg	4.17	4.09	4.04	4.34	0.13	0.66	0.72
Average weight on day 21, kg	6.17	6.03	5.99	6.30	0.18	0.79	0.81
Average daily gain (D0 to D21), g	213.8	245.4	231.7	238.3	5.18	0.18	0.18

**Table 2:** Effects of KEH on antioxidant capacity in serum of sows and piglets.

Items	Levels				SEM	*p*-Value	
	0%	0.75%	1.50%	3%		Linear	Quadratic
Umbilical cord blood							
T-SOD, U/mL	156.7	165.0	160.2	167.1	2.78	0.10	0.27
MDA, nmol/mL	2.68	2.56	2.63	1.90	0.14	0.22	0.45
CAT, U/mL	7.40	8.53	7.81	8.36	0.15	0.18	0.38
GSH-Px, U/mL	188.3	219.4	238.8	271.5	10.90	0.02	0.08
Piglet serum							
Day 7							
T-SOD, U/mL	118.0	121.7	137.1	134.1	2.81	0.30	0.46
MDA, nmol/mL	4.39	4.13	4.00	4.26	0.17	0.76	0.71
CAT, U/mL	2.73	2.94	3.98	5.67	0.30	0.14	0.20
GSH-Px, U/mL	307.0	316.0	255.3	281.7	9.20	0.24	0.24
Day 14							
T-SOD, U/mL	123.3	124.1	123.2	124.9	2.36	0.40	0.39
MDA, nmol/mL	4.28	4.05	3.95	3.59	0.17	0.17	0.39
CAT, U/mL	4.45	4.78	4.58	5.21	0.50	0.78	0.94
GSH-Px, U/mL	257.9	265.9	279.7	333.3	11.67	0.21	0.37
Day 21							
T-SOD, U/mL	121.6	130.4	125.6	134.9	2.76	0.11	0.22
MDA, nmol/mL	5.20	4.18	4.93	3.87	0.16	0.11	0.19
CAT, U/mL	4.67	5.82	5.83	4.72	0.26	0.63	0.02
GSH-Px, U/mL	300.2	392.3	318.2	294.9	13.17	0.79	0.12

T-SOD, total superoxide dismutase; MDA, malondialdehyde; CAT, catalase; GSH-Px, glutathione peroxidase.

**Table 3:** Effects of KEH on immunoglobulin concentrations in serum of sows and piglets.

Items	Levels				SEM	*p*-Value	
	0%	0.75%	1.50%	3%		Linear	Quadratic
Umbilical cord blood, g/L							
IgA	0.96	1.25	1.58	1.21	0.07	0.08	0.01
IgM	0.89	0.97	0.93	0.91	0.04	0.22	0.02
IgG	0.57	0.85	0.62	0.65	0.16	0.89	0.84
Piglet serum, g/L							
Day 7							
IgA	1.15	1.48	1.42	1.36	0.07	0.14	0.34
IgM	1.06	1.09	1.07	1.01	0.02	0.51	0.29
IgG	7.70	7.90	8.01	5.91	0.18	0.02	<0.01
Day 14							
IgA	1.02	1.08	1.18	1.37	0.07	0.07	0.20
IgM	1.01	1.04	1.00	1.11	0.02	0.15	0.18
IgG	8.11	8.28	8.31	7.29	0.29	0.41	0.50
Day 21							
IgA	1.00	1.21	1.37	1.18	0.09	0.14	0.25
IgM	0.94	1.13	1.14	1.07	0.04	0.02	0.04
IgG	5.68	7.07	7.12	7.79	0.22	0.01	0.04

IgA, immunoglobulin A; IgM, immunoglobulin M; IgG, immunoglobulin G.

**Table 4:** Effects of KEH on thyroid hormones and inflammatory cytokine levels in serum of piglets.

Items	Levels				SEM	*p*-Value	
	0%	0.75%	1.50%	3%		Linear	Quadratic
Day 7							
T3, ng/mL	0.73	1.13	0.86	0.98	0.07	0.27	0.03
T4, ng/mL	45.59	62.88	49.85	55.25	3.43	0.52	0.37
IL-1β, pg/mL	16.60	11.50	11.61	16.89	0.50	0.91	<0.01
IL-6, pg/mL	56.92	50.56	55.37	54.37	1.02	0.79	0.52
TNF-α, pg/mL	117.3	115.8	114.8	107.9	2.52	0.19	0.38
Day 14							
T3, ng/mL	0.84	1.01	0.84	0.94	3.41	0.73	0.78
T4, ng/mL	56.00	70.22	66.71	69.03	3.31	0.60	0.58
IL-1β, pg/mL	22.65	18.19	18.37	13.01	0.89	<0.01	0.02
IL-6, pg/mL	54.56	46.03	56.80	53.01	1.47	0.72	0.77
TNF-α, pg/mL	103.6	102.9	95.6	100.2	1.11	0.15	0.23
Day 21							
T3, ng/mL	0.44	0.79	0.52	0.53	0.04	0.55	0.54
T4, ng/mL	59.56	57.47	51.72	54.84	3.41	0.96	0.89
IL-1β, pg/mL	22.32	13.16	13.12	10.93	0.30	<0.01	<0.01
IL-6, pg/mL	79.66	55.87	55.19	65.21	2.32	0.18	<0.01
TNF-α, pg/mL	101.4	97.5	94.7	111.4	2.70	0.33	0.13

T3, triiodothyronine; T4, thyroxine; IL-1β, interleukin-1β; IL-6, interleukin-6; IL-10, interleukin-10; TNF-α, tumor necrosis factor-α.

**Table 5:** Effects of KEH on the contents of short-chain fatty acids in feces (day 21) of sows and piglets.

Items	Levels				SEM	*p*-Value	
	0%	0.75%	1.50%	3%		Linear	Quadratic
Sows, mg/g							
Acetic acid	3.35	3.62	3.87	3.07	0.14	0.16	0.09
Propionic acid	1.36	1.57	1.36	1.24	0.09	0.72	0.27
Isobutyric acid	0.43	0.47	0.44	0.56	0.04	0.16	0.28
Butyric acid	1.69	1.65	2.07	1.74	0.19	0.13	0.21
Isovaleric acid	1.11	1.04	1.04	1.33	0.10	0.16	0.38
Valeric acid	0.37	0.57	0.43	0.69	0.04	0.48	0.78
Total short-chain fatty acids	7.73	8.40	8.35	9.02	0.39	0.12	0.16
Piglets, mg/g							
Acetic acid	6.73	7.83	6.92	6.23	0.32	0.88	0.31
Propionic acid	3.74	4.20	3.75	3.92	0.26	0.57	0.50
Isobutyric acid	0.35	0.50	0.51	0.40	0.02	0.65	0.06
Butyric acid	1.40	1.82	1.86	1.76	0.08	0.23	0.13
Isovaleric acid	0.53	0.89	0.78	0.64	0.05	0.72	0.07
Valeric acid	0.32	0.45	0.42	0.34	0.02	0.90	0.02
Total short-chain fatty acids	12.78	15.69	14.71	13.70	0.77	0.75	0.27

**Table 6:** Chemical composition of KEH.

Chemical Composition	Content
Alginate Polysaccharide, %	4.00
Alginate Oligosaccharide (Calculated as Galacturonic Acid), %	7.99
Betaine, mg/kg	829.0
Iodine, mg/kg	1164
Potassium (Calculated as K_2_O), %	17.47
Phosphorus (Calculated as P_2_O_5_), %	22.11
Moisture, %	2.33
Crude Ash, %	61.00
Organic Matter (Dry Basis), %	27.00
Crude Fiber, %	0.90
Crude Fat, g/kg	17.00
Crude Protein, %	3.78
Aspartic Acid, %	0.38
Threonine, %	0.11
Serine, %	0.10
Glutamic Acid, %	0.48
Proline, %	0.03
Glycine, %	0.13
Alanine, %	0.18
Cystine, %	0.07
Valine, %	0.10
Methionine, %	0.03
Isoleucine, %	0.06
Leucine, %	0.09
Tyrosine, %	0.09
Phenylalanine, %	0.15
Histidine, %	0.07
Lysine, %	0.12
Arginine, %	0.08
Tryptophan, %	0.00

**Table 7:** Ingredients and nutrient levels of basal diets of sows during late gestation and lactation (as-fed basis).

Items	Late Gestation	Lactation
Ingredient, %		
Corn	61.71	61.94
Soybean meal	17.5	15.5
Wheat bran	8.1	-
Extruded soybean	-	5
Beet pulp	8.0	6.3
Fish meal	-	3
Soybean oil	0.8	1.3
Premix ^1^	4.0	4.0
Salt	0.35	0.4
Nutrient Levels (Calculated values)		
Digestible energy, MJ/kg DM	13.31	13.95
Metabolizable energy, MJ/kg DM	12.80	13.43
Crude protein, %	14.91	16.68
Ether extract, %	3.79	5.24
Crude fiber, %	4.59	3.84
Calcium, %	0.85	0.90
Available phosphorus, %	0.38	0.47

^1^ Premix provided per kilogram of complete feed: vitamin A, 240–300 KIU; vitamin D3, 50–125 KIU; vitamin E, 500 IU; vitamin K3, 45 mg; vitamin B1, 50 mg; vitamin B2, 150 mg; vitamin B6, 100 mg; vitamin B12, 0.5 mg; niacin, 650 mg; pantothenic acid, 450 mg; folic acid, 80 mg; biotin, 10 mg; Fe, 2.4–18 g; Cu, 0.2–0.62 g; Zn, 1–2.5 g; Mn, 0.5 2 g; I, 10–50 mg; Se, 5 12.5 mg; calcium, 80 g; phosphorus, 60 g; choline chloride, 10 mg; water, 10%.

## Data Availability

The original contributions presented in this study are included in the article and Supplementary Materials. Further inquiries can be directed to the corresponding authors.

## Supplementary Materials

The following supporting information can be downloaded at: https://www.mdpi.com/article/10.3390/md24100354/s1. Figure S1: Effect of dietary KEH supplementation on backfat thickness at postpartum 24 hours of sows. Figure S2: Effect of dietary KEH supplementation on live born size per litter of sows. Figure S3: Effects of KEH on antioxidant capacity in serum of sows and piglets. a. Effect of dietary KEH supplementation on GSH-Px of umbilical cord blood; b. Effect of dietary KEH supplementation on CAT of piglets on day 21. Figure S4: Effects of KEH on immunoglobulin concentrations in serum of sows and piglets. a. Effect of dietary KEH supplementation on IgA of umbilical cord blood; b. Effect of dietary KEH supplementation on IgM of umbilical cord blood; c. Effect of dietary KEH supplementation on IgG of piglets on day 7; d. Effect of dietary KEH supplementation on IgM of piglets on day 21; e. Effect of dietary KEH supplementation on IgG of piglets on day 21. Figure S5: Effects of KEH on thyroid hormones and inflammatory cytokine levels in serum of piglets. a. Effect of dietary KEH supplementation on T3 of piglets on day 7; b. Effect of dietary KEH supplementation on IL-1β of piglets on day 7; c. Effect of dietary KEH supplementation on IL-1β of piglets on day 14; d. Effect of dietary KEH supplementation on IL-1β of piglets on day 21; e. Effect of dietary KEH supplementation on IL-6 of piglets on day 21. Figure S6: Effect of dietary KEH supplementation on valeric acid of piglets on day 7.

## Abbreviations

The following abbreviations are used in this manuscript:

ADFI	average daily feed intake
ADG	average daily gain
BF	backfat thickness
BW	body weight
C2	acetic acid
C3	propionic acid
C4	butyric acid
C5	valeric acid
CAT	catalase
GSH-Px	glutathione peroxidase
i-C4	isobutyric acid
i-C5	isovaleric acid
IgA	immunoglobulin A
IgG	immunoglobulin G
IgM	immunoglobulin M
IL-10	interleukin-10
IL-1β	interleukin-1β
IL-6	interleukin-6
KEH	kelp enzymatic hydrolysate
MDA	malondialdehyde
OTUs	operational taxonomic units
ROS	reactive oxygen species
SCFAs	short-chain fatty acids
T3	triiodothyronine
T4	thyroxine
TNF-α	tumor necrosis factor-α
TSCFAs	total short-chain fatty acids
T-SOD	total superoxide dismutase
