# Supplementary material for: Effects of Dietary Kelp Enzymatic Hydrolysate Supplementation on Performance, Antioxidant Capacity, Immune Function, and Intestinal Health of Sows and Their Piglets
Source: Mar Drugs. 2026 Oct 9;24(10):354. doi: 10.3390/md24100354 (PMC13706102; doi:10.3390/md24100354)
Supplement: Supplementary file 1 [file marinedrugs-24-00354-s001.zip › marinedrugs-4516843-supplementary-final version.pdf]

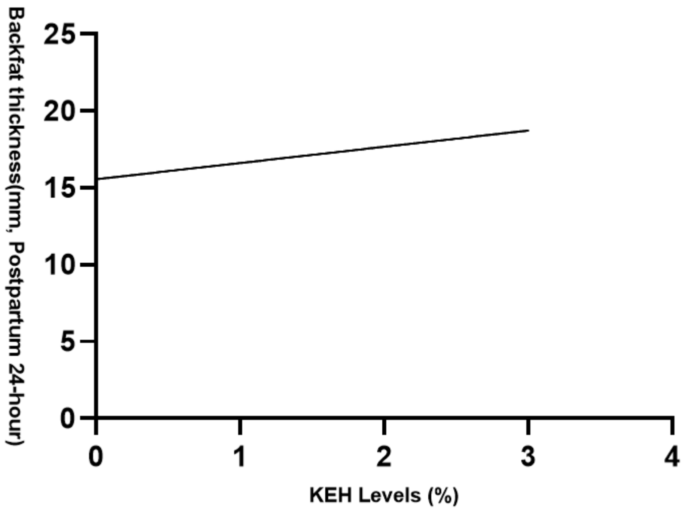

Figure S1. Effect of dietary KEH supplementation on backfat thickness at postpartum 24 h of sows.

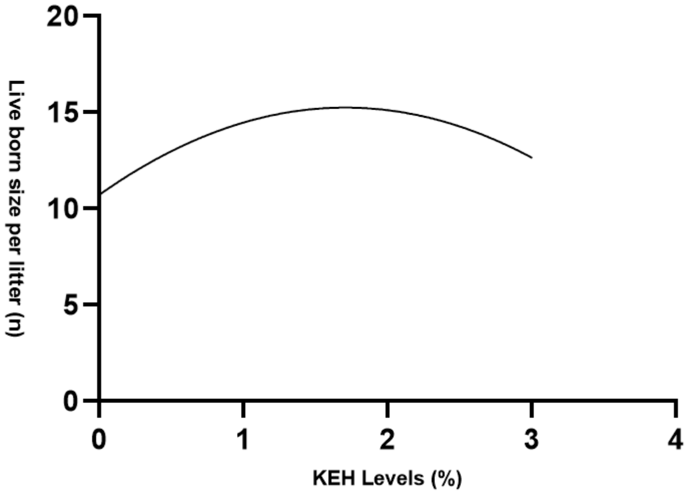

Figure S2. Effect of dietary KEH supplementation on live born size per litter of sows.

a

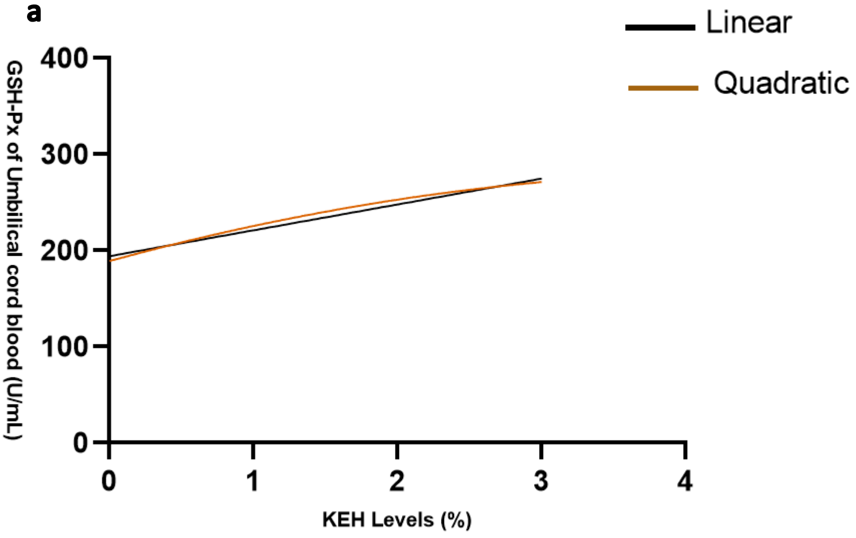

b

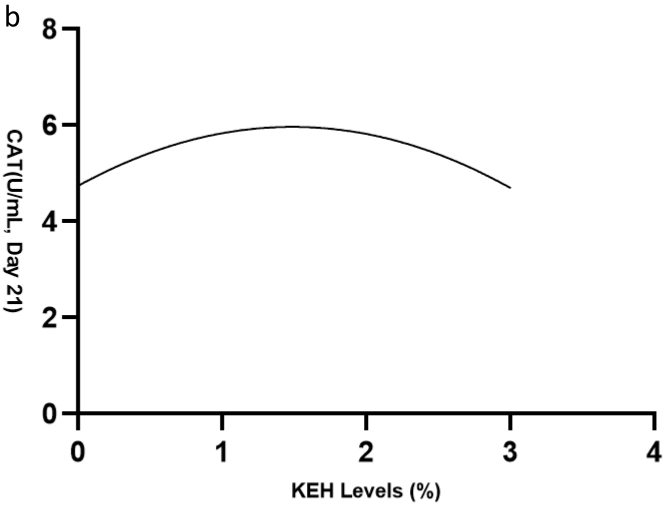

Figure S3. Effects of KEH on antioxidant capacity in serum of sows and piglets

a. Effect of dietary KEH supplementation on GSH-Px of umbilical cord blood.

b. Effect of dietary KEH supplementation on CAT of piglets on day 21.

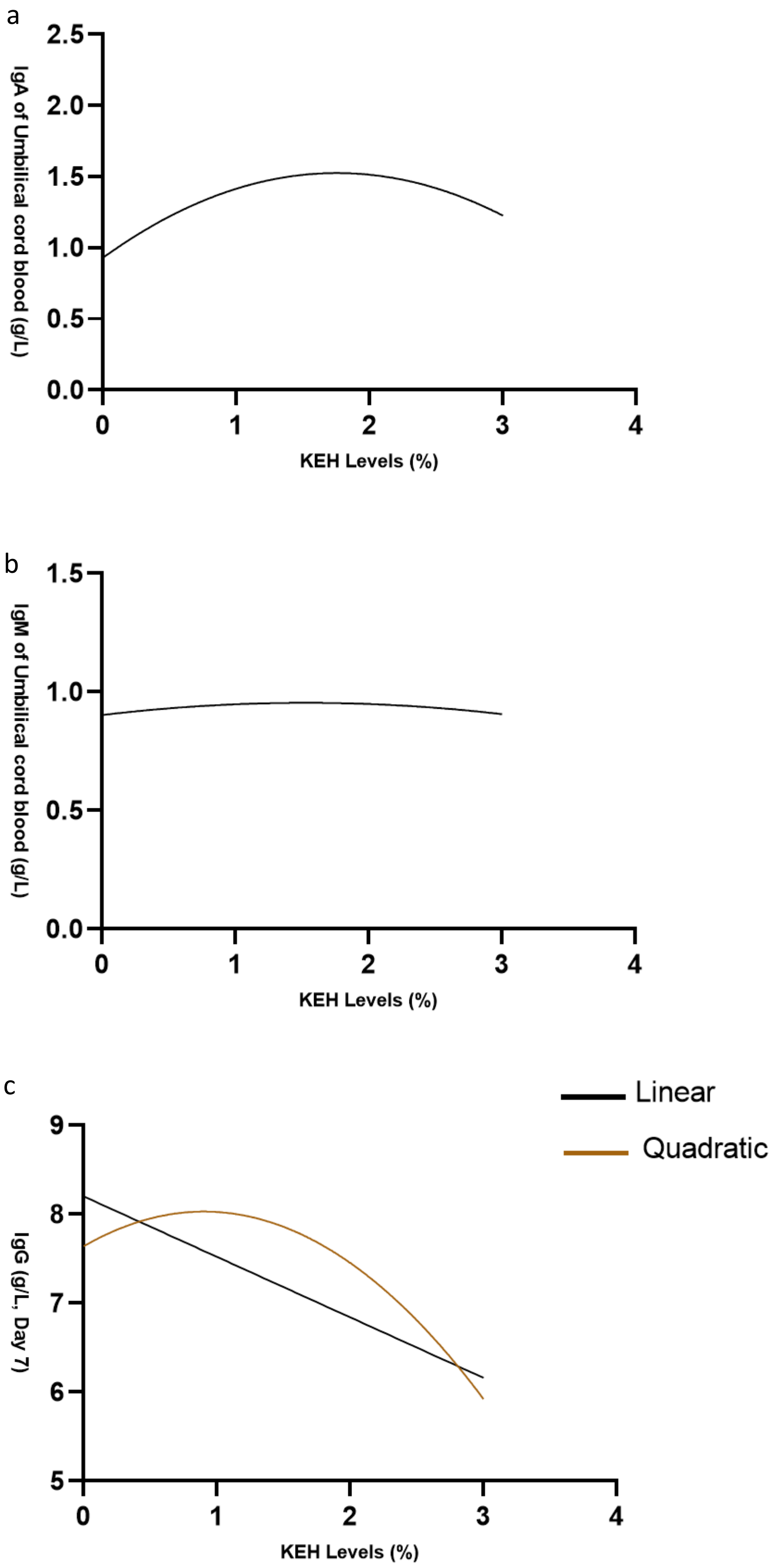
a
IgA of Umbilical cord blood (g/L)
2.5
2.0
1.5
1.0
0.5
0.0
0
1
2
3
4
KEH Levels (%)
b
IgM of Umbilical cord blood (g/L)
1.5
1.0
0.5
0.0
0
1
2
3
4
KEH Levels (%)
c
Linear
Quadratic
IgG (g/L, Day 7)
9
8
7
6
5
0
1
2
3
4
KEH Levels (%)

d

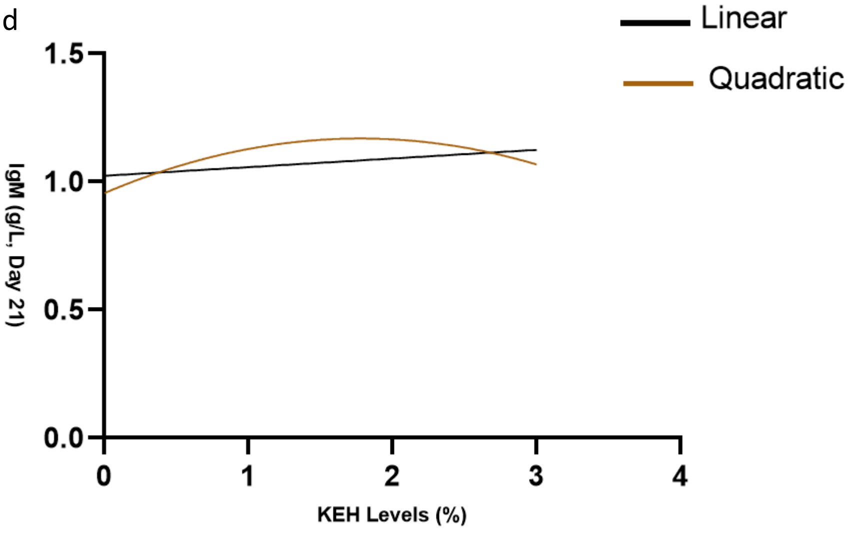

e

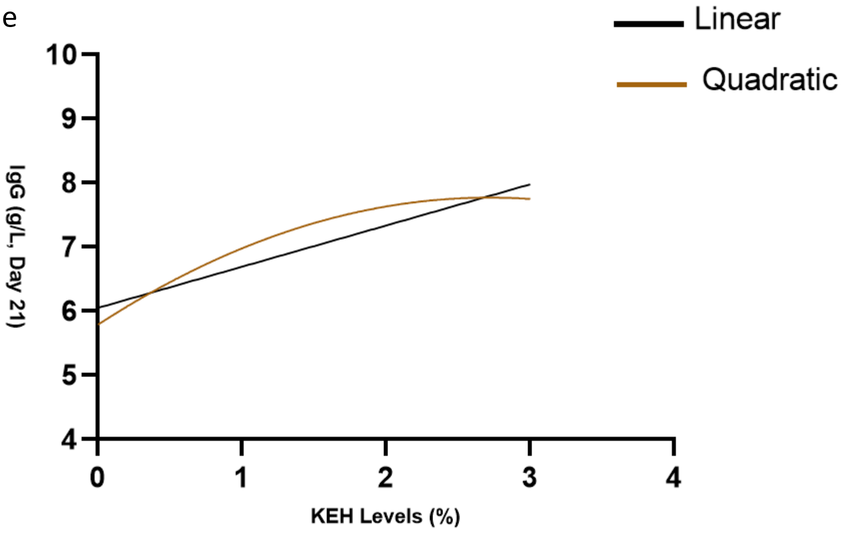

Figure S4. Effects of KEH on immunoglobulin concentrations in serum of sows and piglets.

a. Effect of dietary KEH supplementation on IgA of umbilical cord blood.

b. Effect of dietary KEH supplementation on IgM of umbilical cord blood.

c. Effect of dietary KEH supplementation on IgG of piglets on day 7.

d. Effect of dietary KEH supplementation on IgM of piglets on day 21.

e. Effect of dietary KEH supplementation on IgG of piglets on day 21.

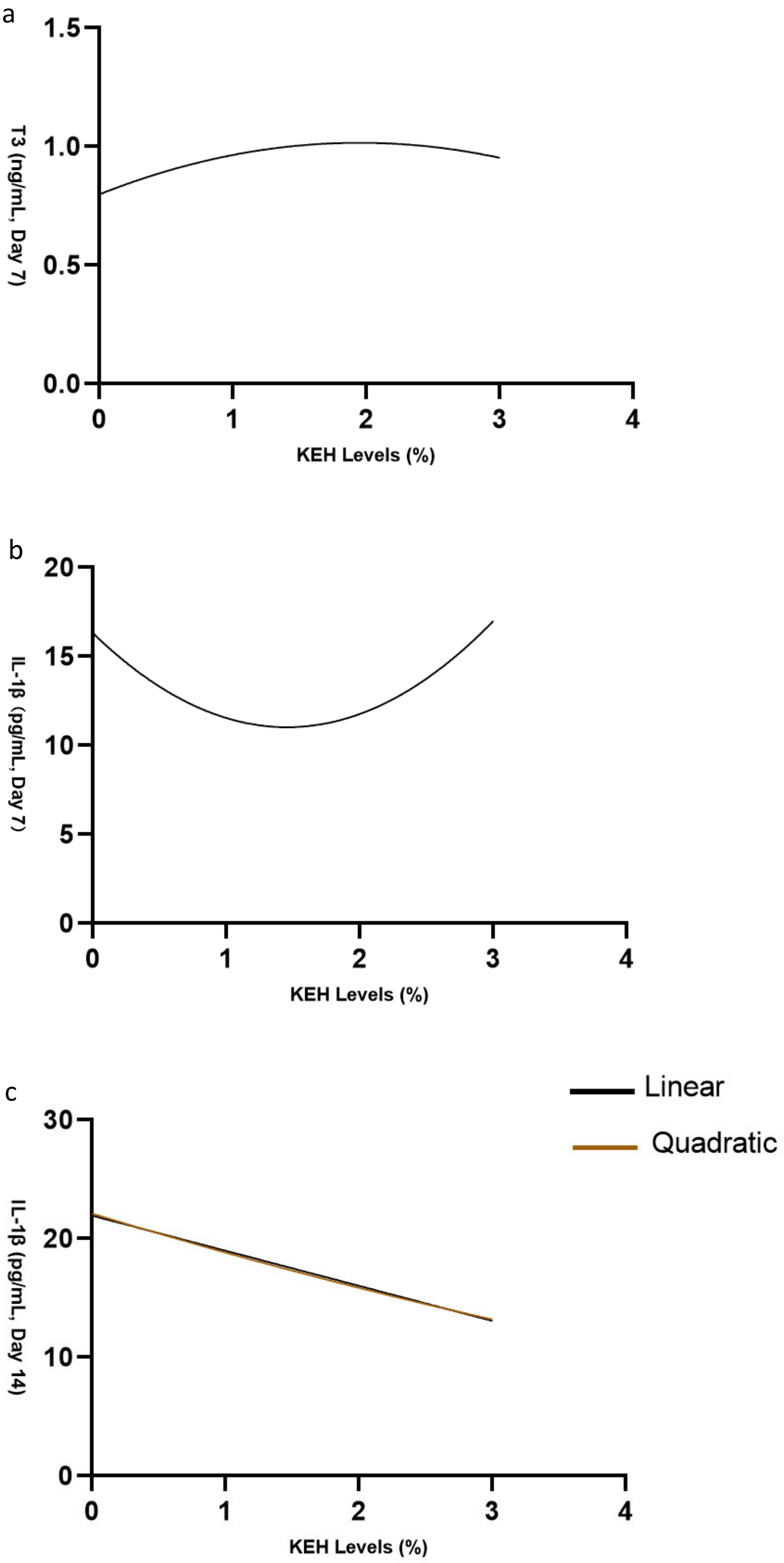
a
1.5
1.0
0.5
0.0
T3 (ng/mL, Day 7)
0
1
2
3
4
KEH Levels (%)
b
20
15
10
5
0
IL-1β (pg/mL, Day 7)
KEH Levels (%)
c
Linear
Quadratic
30
20
10
0
IL-1β (pg/mL, Day 14)
KEH Levels (%)

d

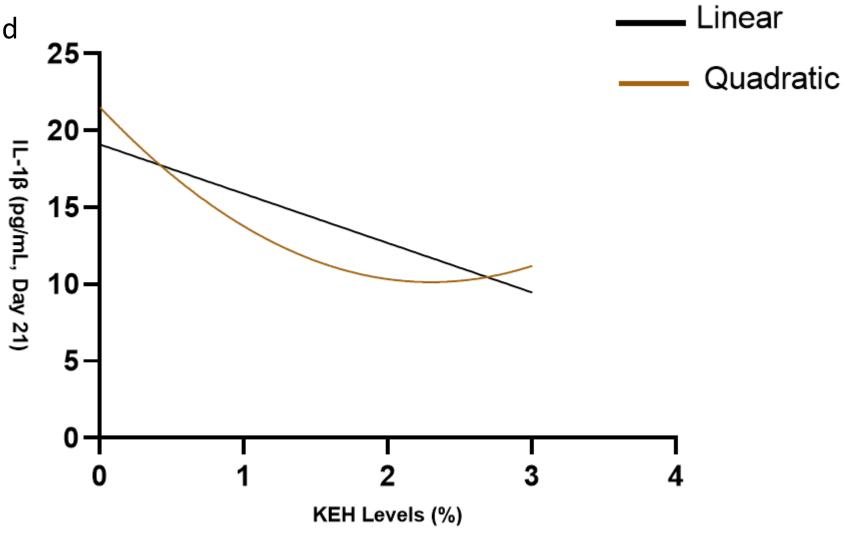

e

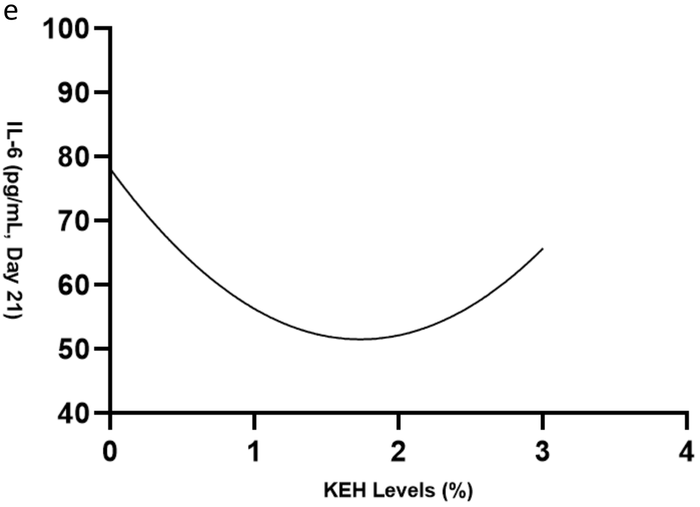

Figure S5. Effects of KEH on thyroid hormones and inflammatory cytokine levels in serum of piglets

a. Effect of dietary KEH supplementation on T3 of piglets on day 7.

b. Effect of dietary KEH supplementation on IL-1β of piglets on day 7.

c. Effect of dietary KEH supplementation on IL-1β of piglets on day 14.

d. Effect of dietary KEH supplementation on IL-1β of piglets on day 21.

e. Effect of dietary KEH supplementation on IL-6 of piglets on day 21.

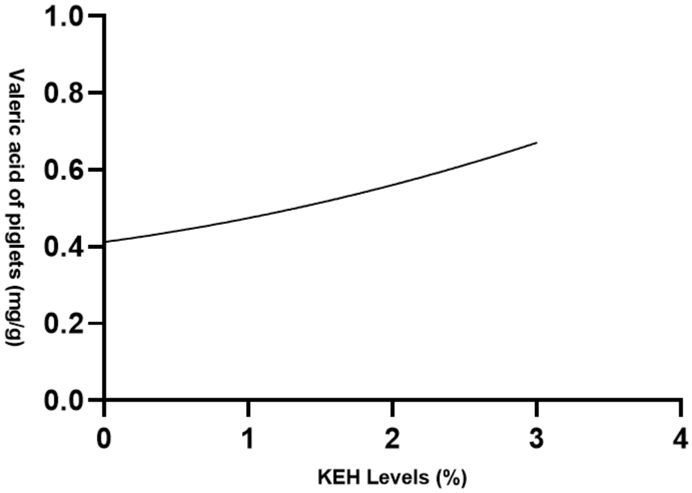

Figure S6 Effect of dietary KEH supplementation on valeric acid of piglets on day 7.
